# 1818. A Network Meta-Analysis of Antibiotic Efficacy for the Treatment of Skin and Soft Tissue Infections

**DOI:** 10.1093/ofid/ofad500.1647

**Published:** 2023-11-27

**Authors:** Marianne Go-Wheeler, Sarah C Mann, Sindalisa Hean, Matthew H Schwartz, Ann Pham, Stephen Judge, Amy Y Kang, Pamela Lee, Raul Macias Gil, Bryn M Launer, Li Tao, Elliot I Miller, Evelyn A Flores, Megan H Nguyen, Maddie Yeh, Christiana Speciale, Loren G Miller

**Affiliations:** Division of Infectious Diseases, Department of Medicine, Harbor-UCLA Medical Center, Torrance, California; Division of Infectious Diseases, University of Colorado, Aurora, CO, Aurora, Colorado; Division of Infectious Diseases, Harbor-UCLA Medical Center, Torrance, CA USA, Torrance, California; Medical College of Georgia, Augusta, GA, USA, Augusta, Georgia; Division of Infectious Diseases, Kaiser Permanente South Bay Medical Center, Harbor City, CA USA, Harbor City, California; Harbor-UCLA Medical Center, Torrance, California; Chapman University School of Pharmacy, Irvine, CA, Irvine, California; Division of Infectious Diseases, Department of Medicine, Harbor-UCLA Medical Center, Torrance, CA USA, Torrance, California; Harbor-UCLA Medical Center, Torrance, California; Lundquist Institute at Harbor-UCLA, Torrance, CA USA, Torrance, California; Department of Medicine, Harbor-UCLA Medical Center, Torrance, CA USA, Torrance, California; Stanford University, Stanford, CA, USA, Stanford, California; Division of Infectious Diseases, the Lundquist Institute at Harbor-UCLA Medical Center, Torrance, CA, Torrance, California; University of California Irvine, Irvine, California; University of California, Berkley, Berkeley, CA, Berkley, California; University of California Irvine, Irvine, CA, USA., Irvine, California; Lundquist Institute at Harbor-UCLA Medical Center, Los Angeles, California

## Abstract

**Background:**

Skin and soft tissue infections (SSTIs) are very common bacterial infections. Numerous clinical trials have compared antibiotics for SSTI treatment. However, trials typically have non-inferiority designs that give little insight as to which antibiotic classes have superior efficacy.

**Methods:**

We performed a systematic literature review and a network meta-analysis of published SSTI treatment trials. Using standardized key words, we searched PubMed and Embase for SSTI clinical trials published from 1/1/66 to 5/31/22. We excluded trials on diabetic foot infections, non-generalizable populations (e.g., burn associated), and studies with outcomes not involving clinical resolution or cure. Abstracts with relevant clinical trial data were pulled and, if inclusion criteria met, had manuscript data abstracted. Two reviewers independently performed abstract and manuscripts reviews and data abstraction. For the network meta-analyses, the comparator antibiotic class was glycopeptides. Two analyses were performed using intention to treat (ITT) and clinically evaluable (CE) populations. Clinical trial quality was measured using the PEDro score.

**Results:**

We reviewed 5,469 abstracts, of which 260 were pulled for data abstraction and 93 contained analyzable trial data (Fig. 1). Clinical trial quality varied widely (PEDro score range 2-10, median 8). We identified 26 analyzable antibiotic classes or combinations for comparison. In the ITT model, cure rate for oxazolidinones (OR 1.28 [95% CI 1.10-1.49]), flucloxacillin plus clindamycin (OR 1.61 [95% CI 1.03-2.53]), lipopeptides (OR 1.66 [95% CI 1.04-2.66]), and tetracyclines OR 1.69 [95% CI 1.24-2.30]), significantly differed from glycopeptides. (Fig. 2) In the CE model, cure rate for oxazolidinones (OR 1.29 [95% CI 1.01-1.64]) and tetracyclines (OR 2.05 [95% CI 1.37-3.06]) significantly differed.

**Figure:**
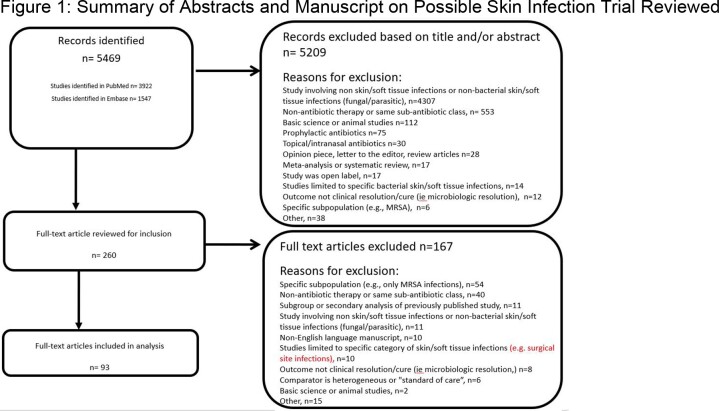

**Figure:**
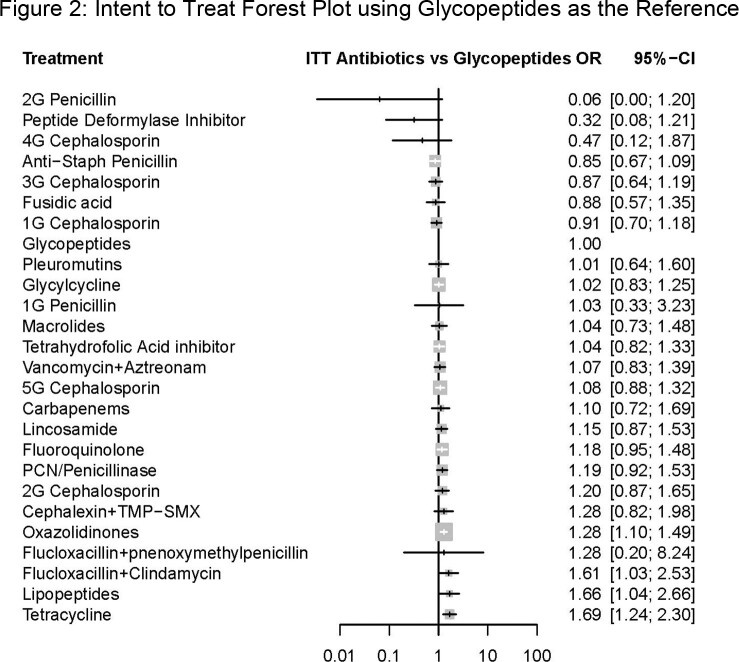

**Conclusion:**

We found that most (22/26) antibiotic classes or combinations used for SSTI treatment had similar efficacy to glycopeptides. However, oxazolidinones, lipopeptides, flucloxacillin plus clindamycin, and tetracyclines may have superior efficacy compared to glycopeptides. Treatment guidelines may wish to favor these latter classes for patients at high risk of treatment failure.

**Disclosures:**

**Amy Y. Kang, Pharm.D., BCIDP**, Paratek: Grant/Research Support **Elliot I. Miller, BS**, Epic Systems, Verona, WI: Employee **Loren G. Miller, MD MPH**, ContraFect: Grant/Research Support|GSK: Grant/Research Support|Medline: Grant/Research Support|Merck: Grant/Research Support|Paratek: Grant/Research Support

